# Evaluating the Correlation between Ptosis Improvement and Immediate Postoperative Lagophthalmos following Blepharoptosis Surgery under General Anesthesia in Pediatric Patients

**DOI:** 10.3390/jcm13041173

**Published:** 2024-02-19

**Authors:** Jae Hoon Jeong, Chong Soo Park

**Affiliations:** 1 Department of Plastic and Reconstructive Surgery, Seoul National University Bundang Hospital, Seongnam 13620, Republic of Korea; drj2h@snubh.org; 2 Department of Plastic and Reconstructive Surgery, Seoul National University College of Medicine, Seoul 03080, Republic of Korea

**Keywords:** ptosis, blepharoptosis, lagophthalmos, MRD1 (marginal reflex distance 1), general anesthesia

## Abstract

**Background**: The objective of this study is to establish a potential correlation between postoperative improvement in upper eyelid ptosis, measured by the marginal reflex distance 1 (MRD1), and the immediate postoperative degrees of lagophthalmos (IPDL). The established correlation is then used to assess whether IPDL can be used as a reliable indicator of successful treatment of eyelid ptosis. **Methods**: This retrospective study involved 19 patients, aged 1 to 11, with a total of 28 eyes affected by ptosis, who underwent surgery under general anesthesia at Seoul National University Bundang Hospital from January 2018 to December 2022. We monitored the MRD1 of the patients for over six months postoperatively and measured the IPDL. **Results**: After ptosis correction surgery, no statistically significant correlation was observed between the improvement in MRD1 and IPDL. Furthermore, the degree of postoperative eyelid ptosis improvement was found to vary and was not consistently sustained, attributable to a range of factors. **Conclusions**: Our study did not establish a statistically significant correlation between IPDL and ptosis improvement as measured by MRD1. Further research is needed to draw definitive conclusions about their correlation.

## 1. Introduction

Ptosis, characterized by drooping of the upper eyelid, can be congenital (present at birth) or acquired (developing later in life), each with various causes. It is a prevalent condition affecting individuals across all age groups. In Korea, the prevalence of ptosis is approximately 13.5% [1]. The prevalence of ptosis in the pediatric population is also quite significant, even though the exact figures vary. In the United States, a study indicated an incidence rate of 7.9 per 100,000 individuals under the age of 19 years, with a 95% confidence interval of 6.4 to 9.5 [2]. Of these young patients with ptosis, 89.7% (96 patients) were diagnosed with congenital-onset disease. Furthermore, 75% (81 patients) of these cases were identified as simple congenital ptosis, corresponding to a birth prevalence of 1 in 842 [2].

The impact of ptosis extends beyond the physical appearance, particularly in pediatric patients [3]. Children with noticeable ptosis may experience psychosocial effects, including decreased self-esteem and social stigmatization, which underscores the importance of timely and effective treatment [4]. Furthermore, ptosis has a potential risk to the developmental trajectory of visual function. Delaying the treatment of significant ptosis can lead to amblyopia, a condition that can cause permanent vision impairment, astigmatism, and strabismus [5,6,7]. The complexity of this condition in children stems from varied etiologies ranging from congenital malformations, neurological disorders, to traumatic injuries, each necessitating a tailored approach for its management [8]. The variations in the etiology necessitate a thorough diagnostic approach to tailor effective treatment plans.

In the realm of ptosis management, one study indicates a preference for operating before the age of four, to reduce the rate of recurrence [9]. While the execution of surgery under local anesthesia is beneficial, permitting an intraoperative assessment of eyelid functionality, this approach is not feasible in pediatric cases. Nonetheless, to mitigate the risk of amblyopia and to minimize ptosis recurrence, early surgical intervention is advocated.

The surgical correction of ptosis in pediatric patients presents challenges, especially when the patient’s age or level of cooperation makes the use of local anesthesia impractical. In such scenarios, general anesthesia becomes indispensable for ensuring a safe and effective surgical environment [10]. However, the use of general anesthesia in pediatric ophthalmic surgery has risks and complications, making the decision-making process all the more critical [11].

Many factors such as preoperative evaluation, operative planning, and postoperative management can affect the outcome of ptosis correction. The anesthetic approach used can also affect the outcome. The choice of anesthesia is predominantly determined by the patient’s ability to cooperate during the surgery rather than by preoperative measurements such as MRD1 or levator muscle function [12]. Specifically, young children, who often cannot cooperate during surgical procedures, typically require general anesthesia.

The surgical methods for ptosis are primarily levator surgery, frontalis sling [13] and frontalis flap surgery. Frontalis flap surgery is also recognized as one of the effective treatments for ptosis. However, this method is not without its drawbacks. While techniques such as endoscopy enable the procedure to be performed through the double eyelid line [14], it typically results in unavoidable scarring near the eyebrow. This scarring can be particularly burdensome for children, often more so than for adults. Consequently, our hospital does not practice this method.

During general anesthesia, the patient cannot be asked to open and close the eye, hence the effectiveness of the ptosis correction procedure cannot be evaluated intraoperatively in pediatric patients [10]. Therefore, in the case of levator surgery, the extent by which the levator muscle is to be pulled and secured to the tarsus is determined before the operation, depending on the degree of ptosis and levator function [15]. In the case of the frontalis sling operation, the immediate postoperative degrees of lagophthalmos (IPDL) to be achieved is also predetermined [12], as the IPDL has been used as a predictive value of ptosis correction [16] when patient cooperation is unattainable. However, it remains unclear whether this correlation between the IPDL and the extent of ptosis correction as measured by MRD1 value translates to the pediatric population.

This study examined ptosis surgery performed under general anesthesia in the pediatric population, focusing on the extent of IPDL which was predetermined based on the preoperative severity of ptosis. Postoperative ptosis improvement was monitored with MRD1 values for a minimum of six months to analyze the relationship between the degree of immediate postoperative lagophthalmos and long-term improvement in ptosis.

## 2. Materials and Methods

The presurgical procedure for ptosis patients is as follows: First, MRD1 is measured to evaluate the degree of ptosis. MRD1 is the distance between the center of the pupillary light reflex and the upper eyelid margin when the eye is in primary gaze and serves as an important indicator for assessing the position and function of the eyelid. This measure is fundamental to patient assessment and surgery choice in facial and ophthalmic plastic surgery [17]. Despite the challenges of obtaining objective measurements due to variables such as the patients raising their eyebrows, the most accurate value is determined by multiple measurements and is recorded in the chart.

Next, a levator function test is conducted. This test evaluates the function of the levator muscle. The range of motion and the strength of the muscle is assessed by having patients move their eyes up and down. However, this test can be challenging if the patient is uncooperative, and it is not performed in pediatric patients under the age of three due to feasibility issues.

Then, the surgical correction of ptosis is determined based on the levator muscle function. The two primary methods are the frontalis sling and the levator advancement surgery. In this study, levator surgery was performed when levator function was higher than or equal to 5 mm and frontalis sling surgery was conducted for levator function less than or equal to 4 mm. For patients under the age of three who were unable to conduct the levator function test, frontalis sling surgery was performed. This approach aligns with other literature, where the frontalis sling is commonly performed in patients with severe ptosis and poor levator function of less than 4 mm [18].

**Figure 1:**
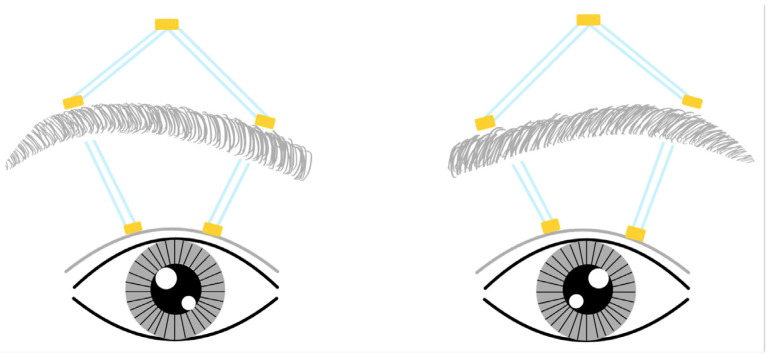
Diagram of the frontalis sling operation method.

The decision to avoid using autogenous fascia lata for frontalis surgery was primarily due to the patients’ young age. Given that the median age was three years, guardians generally preferred the use of a substitute material. Additionally, concerns regarding prolonged surgical duration were taken into account. Furthermore, the supply chain for banked fascia lata was not consistently reliable. All 16 patients undergoing frontalis sling surgery used the FCI ptosis suspension set (ptosis probe by FCI S.A.S, Paris, France). Five 3 mm long incisions were made, two on the upper eyelid margin, two on the upper eyebrow, and one on the forehead, creating a pentagonal shape (Figure 1) where silicone sleeves were tied at the vertex. The levator surgery entailed dissecting the levator aponeurosis and Muller muscle from the underlying conjunctiva and affixing it to the tarsus with 6-0 silk sutures at three points to advance the levator.

In our institution, pediatric patients who underwent surgery for ptosis, especially those with severe ptosis (MRD1 4 mm or more droop [19]) often had accompanying strabismus. Therefore, we collaborated with ophthalmologists specializing in strabismus surgery for treatment. This approach aligns with similar results reported in a case by Srinagesh V. et al. [20].

Pre- and postoperative photographs were taken, and MRD1 values were documented through all examination sessions. The IPDL was also noted. Furthermore, instances of lagophthalmos under general anesthesia prior to surgery were recorded to assess the extent of lagophthalmos induced by ptosis surgery.

After undergoing ptosis surgery, patients are advised to use an ice pack and apply antibiotic ointment twice daily. Additionally, patients were asked to use hyaluronic acid eye drops every two hours and alternate between artificial tear ointments in each eye hourly. This regimen is crucial to prevent dry eyes, which can lead to corneal damage due to lagophthalmos. Postoperative assessments are scheduled for the 5th day, 1st month, and 6 months after the surgery. During these visits, MRD1 check was performed, along with evaluations of lagophthalmos severity and overall eye health, including vision. Follow-up observations were continued for up to 1–2 years to ensure no complications arose.

We performed a retrospective analysis of all pediatric patients that underwent ptosis correction under general anesthesia at Seoul National University Bundang Hospital from January 2018 to December 2022. This retrospective study comprised 53 consecutive patients. Of the 53 patients in this study, 16 were excluded due to the absence of records documenting the IPDL and MRD1 changes both before and after surgery. Additionally, 18 patients who did not have follow-up data exceeding 6 months postsurgery were also excluded. The inclusion and exclusion criteria are clearly delineated in Table 1.

The analysis was subsequently conducted with the remaining 19 patients (encompassing 28 eyes), who had been followed up for over 6 months and for whom measurements of both ptosis degree improvement and MRD1 changes were available. This analysis was carried out using R version 4.1.0 (R Core Team, 2021, Vienna, Austria). 

## 3. Results

In the cohort of the 19 patients that were followed for over 6 months, there were 16 boys and 3 girls. The median age was 3 years old; 14 children were under the age of five, while 4 were over the age of five. The median follow-up period was 14 months. Ten patients had unilateral ptosis while eight had bilateral ptosis. Of the unilateral patients, six underwent surgery exclusively on the right eye and four on the left eye. When both eyes were operated on, each eye was considered individually in the analysis, resulting in a total of 15 cases of right-eye surgeries and 13 of left-eye surgeries. This culminated in a total of 28 surgical cases across the eyes. Table 2 presents a summary of the demographic and clinical characteristics of patients monitored over a six-month period. The surgical techniques utilized were as follows: frontalis sling surgery for 16 patients (24 eyes), and levator surgery for 3 patients (4 eyes).

The comparison of MRD1 values before and after surgery showed a statistically significant difference, as reflected by the *p*-value detailed in Table 3. The median preoperative MRD1 was −1.0 while the median postoperative MRD1 was 1.5, showing a statistically significant improvement. All but two patients showed improvement in MRD1 values, as shown in Table 4. The IPDLs for each patient have also been reorganized in Table 4. To determine the statistical significance of the differences in MRD1 values before and after surgery, the Wilcoxon signed-rank test was employed. The power of the study to detect a significant effect was computed using G*Power 3.1.9.7, developed by Heinrich Heine University, Düsseldorf, which showed an 81% power. As indicated in Table 4, in the 11 cases where the postoperative MRD1 ranged from −2 to 1, indicating an unsatisfactory improvement in ptosis, all underwent reoperation to achieve an MRD1 of 2 or more. However, these cases were excluded from the data for analyzing the correlation between MRD1 and IPDL, as they had not reached the 6-month postoperative follow-up observation.

The final results focused on the IPDL, as well as the degree of improvement in MRD1 values in patients monitored for over six months. As shown in Table 5, the median lagophthalmos change was 5 mm with an IQR of 4 mm to 5.8 mm, a minimum value of 2 mm and maximum of 8 mm. The MRD1 change median was 2.8 m with an IQR of 1.8 mm to 4 mm, a minimum value of 0 mm and maximum of 7 mm.

**Figure 2:**
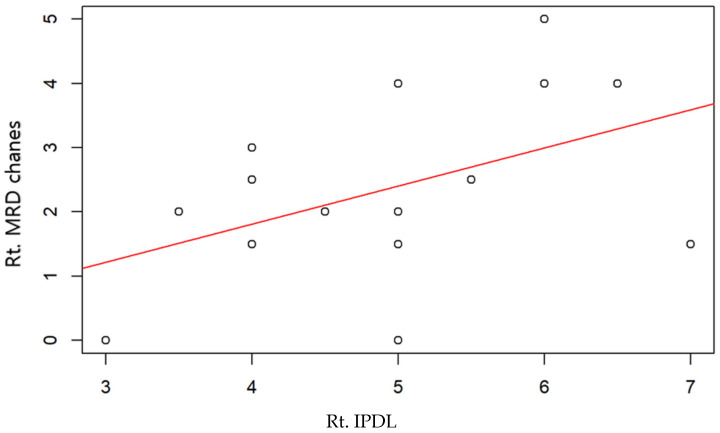
Rt. Eyes scatter plot with regression line. Pearson correlation coefficient 0.46, *p* value < 0.08.

**Figure 3:**
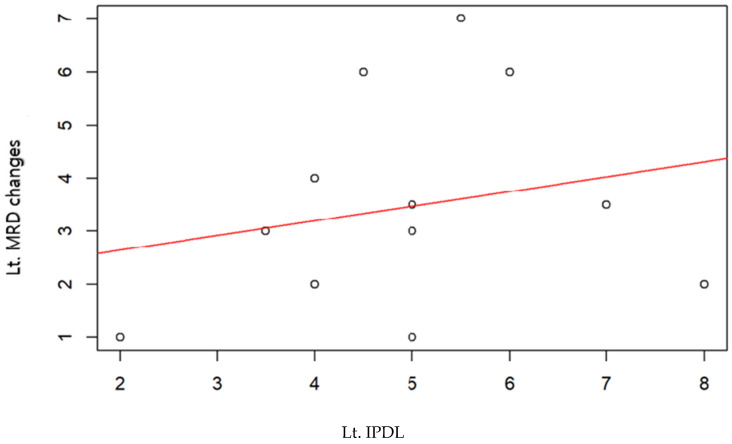
Lt. Eyes scatter plot with regression line. Pearson correlation coefficient 0.23, *p* value < 0.46.

**Figure 4:**
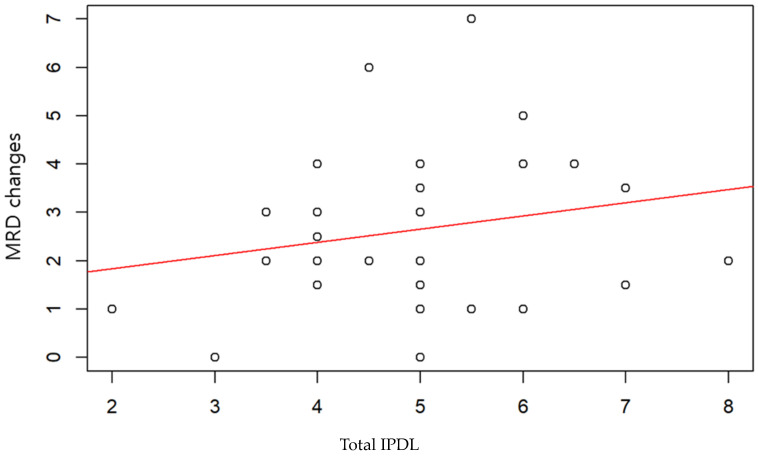
Total scatter plot with regression line. Pearson correlation coefficient 0.21, *p* value < 0.28.

The figure below examines the correlation between the IPDL and the improvement in MRD1 values compared to preoperative levels, observed over 6 months. This analysis was conducted for cases where the right eye (Figure 2), the left eye (Figure 3), and both eyes combined (Figure 4) were operated on, using R version 4.1.0 (R Core Team, 2021). However, none of these cases showed a statistically significant correlation. 

**Figure 5:**
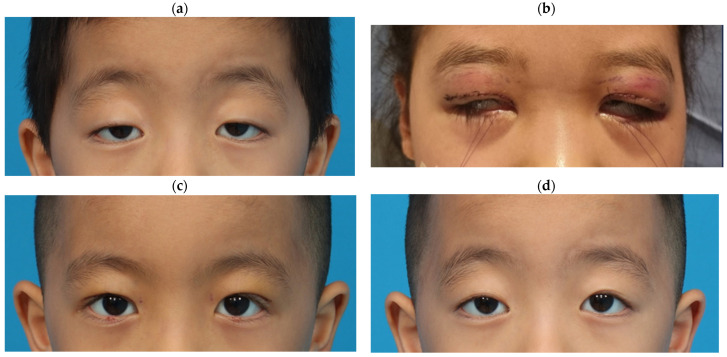
Sample photographs of patients involved in this study. (**a**) Preoperative clinical photograph depicting bilateral ptosis in a 5-year-old boy; (**b**) immediate postoperative photograph showing lagophthalmos in a 9-year-old girl; (**c**) Clinical photograph taken 5 days postoperatively; (**d**) Clinical photograph taken 6 months postoperatively.

Figure 5 illustrates the case of a patient involved in the study. This patient, a 5-year-old boy, underwent surgery under general anesthesia. Levator advancement surgery was performed, as the levator function was higher than 5 mm. A similar procedure was conducted on a 9-year-old girl patient, who displayed lagophthalmos in the immediate postoperative photo. The photographs taken five days and six months postsurgery show significant improvement in ptosis, although the six-month photo indicates a slight undercorrection of the ptosis.

## 4. Discussion

The history of ptosis correction is a testament to the evolution of oculoplastic surgery. Initially, ptosis repair was rudimentary, often involving simple procedures like eyelid shortening. Over the years, the understanding of eyelid anatomy and physiology has significantly improved. Key developments include the introduction of the levator resection technique by Putterman in the 1970s, which allowed for more precise corrections [21]. This was a pivotal moment, shifting the focus towards tailored approaches based on individual anatomical variations and functional needs.

Subsequent advancements in surgical techniques have been driven by a deeper understanding of eyelid dynamics and patient-specific considerations. Innovations like the use of adjustable sutures and endoscopic approaches have provided surgeons with greater control and precision. Today, the field continues to evolve with a focus on minimizing invasiveness while maximizing functional and aesthetic outcomes. There is a study that involved 340 patients with various types of ptosis, where an analysis of different causes and treatments was conducted. This study found similar results across all treatment methods [8]. Consequently, we believe that the difference in outcomes due to surgical methods for improving ptosis may not be significant. Therefore, we primarily used the frontalis sling technique to correct ptosis, and limited use of frontalis flap or levator surgery, considering factors such as the patient’s age and the patient and guardian’s aversion to incision scars. Recent studies have reported that for moderate to severe ptosis, where the function of the levator muscle is less than 4 mm, using the conjoint fascial sheath instead of the traditional frontalis muscle sling surgery has yielded positive results [22].

One of the difficulties in ptosis correction is the uncertainty of the effectiveness of the correction when performed under general anesthesia. Numerous methods have been suggested to resolve this problem, including postoperative lagophthalmos measurement [15,23]. Previous research largely supports the idea that postoperative lagophthalmos plays a pivotal role in determining the success of ptosis surgery. Nevertheless, our study introduces a new perspective, suggesting that the correlation may not be as direct as previously believed. This inconsistency highlights the necessity for additional research in this field to resolve these divergent findings.

The principal aim of this study was to investigate the association between postoperative lagophthalmos and the improvement of ptosis following surgery under general anesthesia. Contrary to expectations of a statistically significant correlation, the results indicated no significant relationship between the postoperative improvement in MRD1 and IPDL. Our findings contest the traditional belief that postoperative lagophthalmos is a dependable indicator of successful ptosis correction [16]. The absence of a significant correlation implies that other elements, perhaps biological or methodological in nature, may have a more critical impact on the outcomes of ptosis surgery than previously recognized.

Consistent with other studies on blepharoptosis, our research also treated surgeries performed on both eyes as distinct procedures, enabling us to analyze the improvement in each eyelid separately [12,15,16,23]. However, this methodology is not without potential inaccuracies. For example, in cases where both eyes exhibit ptosis, asymmetry is common. If surgical correction is focused on the more severely affected eye, the less affected eye might appear undercorrected or could even experience a worsening of symptoms in accordance with Hering’s law [24].

Furthermore, while frontalis sling procedures [25] constitute the majority of surgeries, levator surgery [26] is employed when levator function exceeds 7 mm. In our cohort, a levator function of 5 mm or higher went through levator surgery, and this variation in surgical technique may have influenced the outcomes. It is critical to consider a range of factors to ensure the success of the procedure. Postoperatively, patients who have undergone surgery under general anesthesia often face difficulties in self-managing eye care. Inadequate care, such as rubbing or improper handling of the operated eyes can compromise the maintenance of the desired surgical outcomes [27].

From a clinical standpoint, our results indicate that surgeons should be cautious in depending solely on lagophthalmos as a predictive measurement for the outcomes of ptosis surgery. It is crucial to take into account a wider array of considerations, encompassing individual patient-specific anatomical and physiological attributes, during the planning and execution of ptosis correction procedures.

As mentioned earlier, the outcomes of ptosis surgery can only be evaluated at least six months after the operation. Initially, the desired MRD1 results may be achieved, but changes can occur during the six months. In children who undergo ptosis surgery under general anesthesia, the outcomes frequently do not remain stable as they grow.

Postoperative lagophthalmos typically shows improvement as time progresses and is generally not a major concern [28]. However, if it does occur, diligent eye care, including the use of artificial tears or ointments, is crucial. This is particularly important for young children who are unable to care for themselves, as neglecting this condition can result in severe eye complications. However, this study found that postoperative lagophthalmos, even up to 8 mm, almost completely disappears if proper eye care is administered, indicating that lagophthalmos is generally not a significant issue. A comprehensive understanding of the confluent structures of the orbital septum, levator and conjoint fascial sheath is still a much needed area of research. Since various surgical methods for ptosis are being introduced along with anatomical studies, no single method can be deemed perfect [29]. In this context, it is essential to research and apply various surgical techniques and anatomy to ensure the best outcomes for patients.

This study presents certain limitations that warrant acknowledgment. The sample size, while substantial for a single-center study, remains relatively limited and may not fully represent the diverse range of ptosis cases. The study had less than 80% power to detect a significant effect, as calculated using G*Power 3.1.9.7. Thus, future research should aim to secure a larger sample size. Furthermore, the retrospective design of this study may lead to inherent biases, such as selection and observational biases. Future research would benefit from larger-scale, multicenter studies to improve the generalizability of these findings. Future research endeavors should focus on identifying and quantifying additional predictive factors that contribute to the success of ptosis surgery [30]. Prospective studies, especially those employing a multidisciplinary approach, have the potential to yield more profound insights into the intricate dynamics of ptosis correction surgery. Moreover, the creation of more comprehensive predictive models that include various clinical and anatomical considerations could substantially contribute to advancements in this field.

## 5. Conclusions

Our study did not establish a statistically significant correlation between IPDL and ptosis improvement as measured by MRD1. However, this result cannot confirm the absence of a relationship between IPDL and ptosis improvement as measured by MRD1. Further research is needed to draw definitive conclusions about their correlation.

## Figures and Tables

**Table 1:** Inclusion and exclusion criteria of the retrospective study.

Inclusion Criteria	All pediatric patients that underwent ptosis correction under general anesthesia at Seoul National University Bundang Hospital from January 2018 to December 2022.
Exclusion Criteria	Absence of records documenting the IPDL (immediate postoperative degrees of lagophthalmos).
	Absence of records documenting the MRD1 changes both before and after surgery.
	Absence of follow-up records including MRD1 exceeding 6 months postsurgery.

**Table 2:** Demographics and clinical characteristics of patients.

Patients’ Information		Data
Sex	Male	16
	Female	3
Age (median) (years)		3 (IQR 2, 5) (min 1, max 11)
Follow up periods (months)		14 (IQR 8, 27) (min 7, max 57)
Unilateral	Right	6
	Left	4
Bilateral		9

**Table 3:** Pre- and postoperative MRD1 values.

Preoperative MRD1 (IQR) N = 28 ^1^	Postoperative MRD1 (IQR) N = 28 ^1^	*p*-Value ^2^
−1.0 (−2.3, 0)	1.5 (1.0, 2.1)	<0.001

^1^ Median (IQR); ^2^ Wilcoxon signed-rank test with continuity correction; *p* value < 0.05 was considered statistically significant.

**Table 4:** Supplementary table of pre- and postoperative MRD1 values, MRD1 changes and lagophthalmos changes.

Case No.	Age (Years)	Sex	Eye	Preoperative MRD1	Postoperative MRD1	MRD1 Changes	IPDL
1	2	M	OS	−1	2	3	5
2	4	M	OD	−5	−2	3	4
3	4	M	OS	−5	1	6	4.5
4	3	M	OD	−2	3	5	6
5	3	M	OS	0	3	3	5
6	11	M	OD	2	2	0	3
7	11	M	OS	0.5	1.5	1	2
8	4	M	OD	−3	1	4	5
9	1	M	OD	−1	1	2	3.5
10	1	M	OS	−1	1	2	4
11	11	M	OD	1	2.5	1.5	7
12	11	M	OS	−1	1	2	8
13	3	M	OD	−5	−1	4	6.5
14	1	M	OS	−1	2.5	3.5	5
15	3	M	OD	0	1.5	1.5	5
16	5	F	OD	−2	0.5	2.5	4
17	5	F	OS	−2	2	4	4
18	2	F	OD	−1	3	4	6
19	3	M	OD	−0.5	1.5	2	5
20	3	M	OS	0.5	1.5	1	5
21	3	M	OD	−1	1	2	4.5
22	6	M	OD	−1	−1	0	5
23	3	M	OS	−3	4	7	5.5
24	7	M	OD	−1	1.5	2.5	5.5
25	7	M	OS	−4	2	6	6
26	3	M	OS	0	3	3	3.5
27	2	F	OD	0	1.5	1.5	4
28	2	F	OS	−3	0.5	3.5	7

F, female; M, male; OD, right eye; OS, left eye; MRD1, margin reflex distance 1; IPDL, immediate postoperative degrees of lagophthalmos.

**Table 5:** Pre- and postoperative IPDL and MRD1 changes.

	Lagophthalmos Changes	MRD1 Changes
Median	5	2.8
IQR	4, 5.8	1.8, 4
Min., Max. (mm)	2, 8	0, 7

## Data Availability

The data presented in this study are available on request from the corresponding author. The data are not publicly available due to ethical considerations.
